# Antibacterial Properties of Mesoporous Silica Nanoparticles Modified with Fluoroquinolones and Copper or Silver Species

**DOI:** 10.3390/ph16070961

**Published:** 2023-07-05

**Authors:** Maider Ugalde-Arbizu, John Jairo Aguilera-Correa, Eider San Sebastian, Paulina L. Páez, Estela Nogales, Jaime Esteban, Santiago Gómez-Ruiz

**Affiliations:** 1 Departamento de Química Aplicada, Facultad de Química, Euskal Herriko Unibertsitatea (UPV/EHU), Paseo Manuel Lardizabal 3, 20018 San Sebastián, Spain; 2 Clinical Microbiology Department, IIS-Fundación Jiménez Diaz, UAM, Avenida Reyes Católicos 2, 28040 Madrid, Spain; 3 COMET-NANO Group, Departamento de Biología y Geología, Física y Química Inorgánica, ESCET, Universidad Rey Juan Carlos, C/Tulipán s/n, 28933 Móstoles, Spain; 4 CIBERINFEC-CIBER de Enfermedades Infecciosas, Instituto de Salud Carlos III, 28222 Madrid, Spain; 5 Departamento de Ciencias Farmacéuticas, Facultad de Ciencias Químicas, Universidad Nacional de Córdoba, Unidad de Investigación y Desarrollo en Tecnología Farmacéutica (UNITEFA), Consejo Nacional de Investigaciones Científicas y Técnicas (CONICET), Haya de la Torre y Medina Allende, Ciudad Universitaria, Córdoba X5000HUA, Argentina

**Keywords:** MSN, fluoroquinolone, copper, silver chloride, biofilm

## Abstract

Antibiotic resistance is a global problem and bacterial biofilms contribute to its development. In this context, this study aimed to perform the synthesis and characterization of seven materials based on silica mesoporous nanoparticles functionalized with three types of fluoroquinolones, along with Cu^2+^ or Ag^+^ species to evaluate the antibacterial properties against *Staphylococcus aureus*, *Enterococcus faecalis*, *Escherichia coli*, and *Pseudomonas aeruginosa*, including clinical and multi-drug-resistant strains of *S. aureus* and *P. aeruginosa*. In addition, in order to obtain an effective material to promote wound healing, a well-known proliferative agent, phenytoin sodium, was adsorbed onto one of the silver-functionalized materials. Furthermore, biofilm studies and the generation of reactive oxygen species (ROS) were also carried out to determine the antibacterial potential of the synthesized materials. In this sense, the Cu^2+^ materials showed antibacterial activity against *S. aureus* and *E. coli*, potentially due to increased ROS generation (up to 3 times), whereas the Ag^+^ materials exhibited a broader spectrum of activity, even inhibiting clinical strains of MRSA and *P. aeruginosa.* In particular, the Ag^+^ material with phenytoin sodium showed the ability to reduce biofilm development by up to 55% and inhibit bacterial growth in a “wound-like medium” by up to 89.33%.

## 1. Introduction

At the beginning of the 21st century, the increasing emergence of multi-resistant bacterial pathogens has become a global problem. The World Health Organization (WHO) has already underlined the urgency of designing new antimicrobial molecules because conventional antibiotics are becoming less and less useful as therapeutic agents [1,2]. Antibiotic resistance is closely related to the development of bacterial biofilms because the bacterial cells surrounded by their self-produced biomatrix living inside the biofilm require a higher concentration of antimicrobials than those needed to kill their planktonic counterparts, which can be up to 1000 times higher, making it challenging to develop effective treatments to eradicate bacterial infections [3,4].

In this context, nanomedicine has introduced promising changes in clinical practice that have allowed researchers to address unmet medical needs. In particular, nanoparticles can act as carriers of antibiotics, increasing their ability to penetrate the biofilm bacteria often developed to resist conventional antibiotics [5,6]. Among nanomaterials, silica nanoparticles (MSNs) represent one of the most studied materials in nanomedicine due to their good biocompatibility, good degree of porosity, large surface area, a substantial pore volume, and an interesting ease of surface modification, which allow them to support organic and inorganic compounds as therapeutic agents against bacterial infections [7,8,9,10]. Therefore, in this work, functionalized and fully characterized MSNs were used as therapeutic agents for the prevention of the development of bacterial infections.

In addition, and in the context of the discovery of alternative antibacterial agents, fluoroquinolones (FQ) have been demonstrated to be a broad family of antibiotics that can be widely used against various bacteria, such as *Escherichia coli* or *Pseudomonas aeruginosa*, and have been used since the approval of ciprofloxacin by the Food and Drug Administration (FDA) in 1987 [11,12,13]. However, as the use of FQs has increased, resistance has also emerged as a current problem [14]. Nevertheless, it has been shown that after the incorporation of FQs into MSNs, bacterial susceptibility may increase [15,16].

The use of metals in this biological field is another alternative to the challenge of antibiotic resistance. Metals are impervious to selective resistance [17,18]. In this regard, the literature contains examples of utilizing a formation of coordination compounds with FQ, which has led to the development of improved antibacterial agents [19,20,21,22,23,24,25,26]. Among all metals, Cu^2+^ and Ag^+^ metals constitute excellent alternatives [27,28] owing to their excellent antibacterial activity [29,30,31,32,33].

Thus, to understand the importance of finding adequate therapeutic agents, it is important to note that the occurrence of chronic non-healing infected wounds in developed countries is similar to more visible health problems, such as heart failure (1–2%); therefore, the development of new anti-infectious and epithelial tissue-proliferative treatments is a top medical priority [34].

**Scheme 1:**
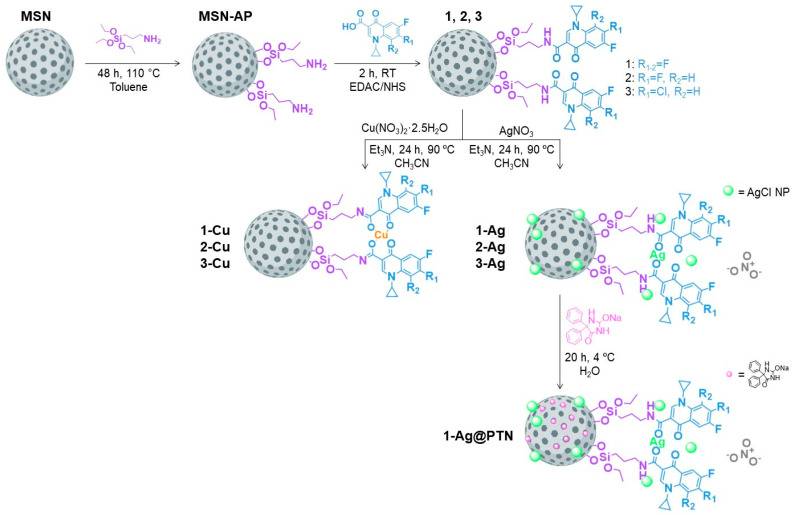
Synthetic routes in the preparation of the functionalized materials.

In this context, the purpose of this work is to develop new antimicrobial molecules and effective treatments against bacterial infections by functionalizing MSN materials with three perhalogenated fluoroquinolone (FQ) derivatives (namely, 3F-FQ, 2F-FQ, and FCl-FQ) based on 1-cyclopropyl-6-fluoro-4-oxo-1,4-dihydroquinolone-3-carboxylic acid (in blue in Scheme 1). Each derivative was synthesized and characterized. Fluoroquinolones have been used in this study because of their high antibacterial potential. In a subsequent step, each material was further functionalized with Cu^2+^ or Ag^+^ species to promote a synergistic antibacterial effect between the metals and fluoroquinolones. Furthermore, our study also involved the adsorption of phenytoin sodium, a well-known proliferative agent [35,36,37], onto one of the silver-functionalized materials, aiming at promoting healing of infected wounds. This proliferative agent demonstrated an additive antibacterial effect when co-administered with silver-containing species, while the phenytoin-loaded silver materials caused an almost complete eradication of *P. aeruginosa* infections in wound-like medium (including components that the synthesized materials would encounter in an in vivo model) and biofilms [38].

In a step toward the development of effective treatments against bacterial infections, in this study, the synthesized materials were tested as broad-spectrum anti-infectious agents against planktonic cultures of commercial strains of *Enterococcus faecalis*, *Escherichia coli*, *Staphylococcus aureus*, and *Pseudomonas aeruginosa*, as well as clinical and multidrug-resistant strains of the latter two. The study also included biofilm studies in both commercial and clinical strains and the determination of reactive oxygen species (ROS) with very promising results that validate future studies with these types of materials in preclinical applications.

## 2. Results

### 2.1. Synthesis and Physicochemical Characterization of Functionalized NPs

**MSN** were functionalized with 3-aminopropyltriethoxysilane to give **MSN–AP** (Scheme 1). Subsequently, these amino-functionalized materials were treated for two hours at room temperature with a 10% solution of the corresponding FQ derivative ligand using an EDAC/NHS coupling reaction to afford **1**, **2**, and **3** (Scheme 1). The incorporation of copper and silver was achieved by the reaction of the material with Cu(NO_3_)_2_·2.5H_2_O or AgNO_3_ under reflux for 24 h, respectively, to obtain **1-Cu**, **2-Cu**, **3-Cu**, and **1-Ag**, **2-Ag**, and **3-Ag** materials (Scheme 1). Finally, the **1-Ag** material was loaded with phenytoin sodium (PTN) in water at a low temperature to achieve the final material, **1-Ag@PTN** (Scheme 1).

#### 2.1.1. Analysis of Size, Morphology, and Textural Properties

**Figure 1:**
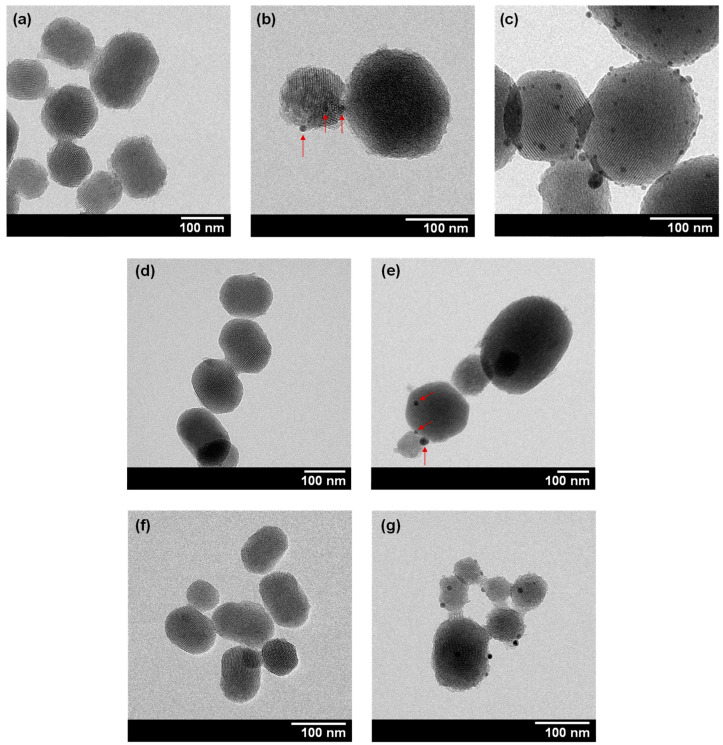
TEM images of (**a**) **1-Cu**, (**b**) **1-Ag**, (**c**) **1-Ag@PTN**, (**d**) **2-Cu**, (**e**) **2-Ag**, (**f**) **3-Cu**, and (**g**) **3-Ag**. Red arrows indicate nanoparticles compatible with Ag nanoparticles.

The synthesized materials were characterized by transmission electronic microscopy (TEM). The micrographs were analyzed using ImageJ^®^ (version 1.53) software to determine the morphology and particle size. As can be seen in Figure 1, in all cases, the nanoparticles show an oval structure with hexagonally arranged porous parallel channels. The particle size in all the cases was in the range of 131–143 nm (for particle size distributions, see Figure S1 in Supporting Information). Compounds **1-Ag**, **2-Ag**, and **3-Ag** showed spherical nanoparticles adhered to MSNs with an average size of 9–12 nm, compatible with Ag nanoparticles.

The starting (**MSN**) and final materials were characterized by nitrogen adsorption/desorption isotherms (BET) in order to determine the most interesting textural features (Table 1), which allowed us to determine whether the materials were loaded with the different agents (Figures S2 and S3 in the Supporting Information provide further details).

In the case of the bare **MSN** nanoparticles, and according to the IUPAC classification, the obtained isotherm can be classified as type IV [39], typical of mesoporous materials [40,41,42,43]. In addition, a hysteresis loop formed between P/P_0_ 0.90–1.0, confirming the capillary condensation processes of the mesoporous materials. The adsorption study showed that the unmodified BET (S_BET_) surface area was 925 m^2^ g^−1^, while after the functionalization reactions, a very significant decrease in the N_2_ adsorption capacity of the final materials was observed. The functionalized systems (namely, **1-Ag**, **1-Ag@PTN**, **2-Cu**, **2-Ag**, **3-Cu**, and **3-Ag**) showed isotherms between type II and type III and the S_BET_ parameters much lower than those of **MSN** (Table 1 and Figure S2), which is indicative of the functionalization occurring inside the pore, inducing a decrease in the pore volume and diameter. Furthermore, PTN adsorption caused **1a-Ag@PTN** to also show a moderate decrease in its N_2_ adsorption capacity (Figure S2, pink lines) compared to the same material without PTN (**1a-Ag**, Figure S2, green lines), reflected in the S_BET_ parameter, which decreased by 35.7% compared to the same material without PTN.

#### 2.1.2. Quantification of the Functionalization Degree by Thermogravimetry and Inductively Coupled Plasma Atomic Emission Spectroscopy

The determination of the functionalization degree of the nanoparticles, estimated from the quantification of the AP, FQ, metal, and PTN content, was performed by thermogravimetry analysis (TGA) and inductively coupled plasma atomic emission spectroscopy (ICP-AES) (Table 2). The amount of mmol of PA, FQ, and PTN per gram of material was determined by TGA, which quantified the mass loss in the temperature range between 120 and 650 °C (Figure S4). The data indicate a functionalization of about 0.70 mmol of AP per gram of **MSN** and between 0.28–0.36 mmol of FQ per gram of material. In addition, the metal-functionalized materials were characterized by ICP-AES to determine the quantity (mmol per g of material) of supported Cu or Ag. The results show that the quantity of Cu was between 0.1–0.2 mmol per gram of material and the amount of silver was between 0.1–0.3 mmol per gram of material (Table 2). Finally, the quantification of phenytoin sodium adsorbed on the **1-Ag@PTN** material was determined by TG, revealing a PTN load of 1.16 mmol per gram of material.

#### 2.1.3. Characterization by Powder X-ray Diffraction Studies

**Figure 2:**
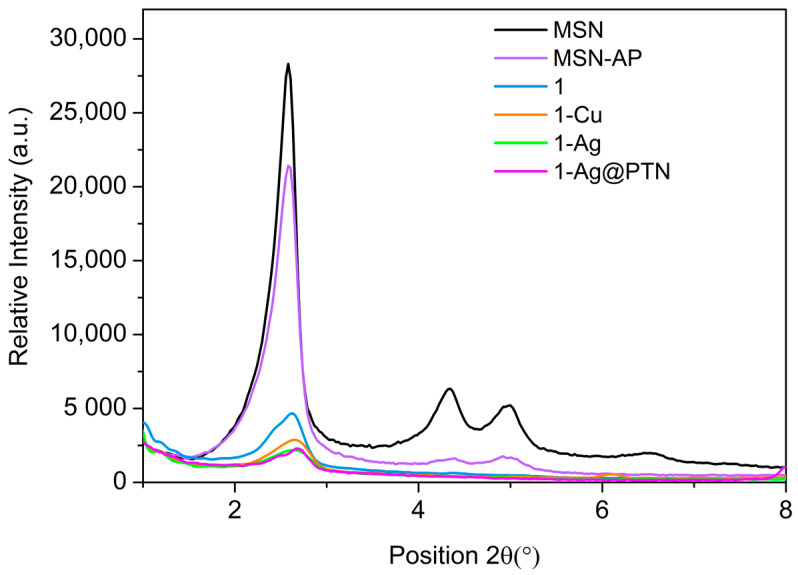
Small-angle PXRD patterns of MSN-based materials.

The starting material, **MSN**, and the corresponding functionalized materials were characterized by powder X-ray diffraction (PXRD) (Figure 2 and Figure S5, Table S1). The unmodified **MSN** materials exhibited three peaks at 2θ of 2.57°, 4.32°, and 4.98°, corresponding to (100), (110), and (200) Miller planes, respectively, which confirmed the mesoscopic order of the material. As can be seen in Figure 2, after the functionalization reactions, the intensity of these peaks (especially of those of planes 110 and 200) decreased due to the partial blocking of the scattering points of the porous system caused by the incorporation of the organic and/or metallic fragments in the system. The diffractograms obtained for the homologous materials based on **2** and **3** had similar patterns (Figure S5).

Furthermore, the presence of AgCl nanoparticles in the silver-containing materials and the correct encapsulation of PTN in the pores of **1-Ag@PTN** were confirmed by large-angle diffractograms (Figures S5 and S6 in the Supporting Information).

The successful functionalization of the materials was further confirmed by Fourier-transformed infrared spectroscopy (FT-IR), diffuse reflectance UV–visible spectroscopy (DR-UV), and solid-state ^13^C CP MAS NMR spectroscopy studies (for details, see the Supporting Information, Figures S7–S9).

### 2.2. In Vitro Studies of Antibacterial Activity

#### 2.2.1. Minimum Inhibitory Concentration (MIC) and Minimal Bactericidal Concentration (MBC)

The antibacterial effects of the materials were evaluated by comparing the MIC and MBC values against two Gram-positive (*S. aureus* ATCC 29213 and *E. faecalis* ATCC 29212) and two Gram-negative (*E. coli* ATCC 25922 and *P. aeruginosa* ATCC 27853) collection strains.

The results collected in Table 3 indicate that the silver-functionalized materials exhibit a broader spectrum of activity compared to the copper-functionalized ones. The former was effective against all four strains, while the latter only demonstrated activity against *S. aureus* and, specifically, against *E. coli*. The observed difference between the antibacterial activity of copper- and silver-functionalized materials could be attributed to the accessibility of the antibacterial agent to its target. Silver nanoparticles are attached to the surface of MSNs and are more accessible compared to copper compounds. This difference in accessibility could contribute to the enhanced antibacterial activity of silver-functionalized materials compared to copper-functionalized systems. Thus, the activity of materials **1-Cu**, **2-Cu**, and **3-Cu** against *S. aureus* and *E. coli* was classified as bactericidal (MBC/MIC < 2). The bactericidal activity shown by these systems against *E. coli* is particularly remarkable, with MIC values of 15.62 µg/mL, lower than that observed in similar systems published previously by our group (31.25–125 µg/mL) [44,45]. Moreover, the MIC values became even more significant in reference to the concentration of copper in the final culture. In that case, the MIC values were between 0.12–0.20 µg/mL, which are somewhat similar or slightly lower than the clinically used drugs [46].

On the other hand, **1-Ag**, **2-Ag**, and **3-Ag** showed activity against all four strains studied, indicative of a broader spectrum of antibacterial activity as compared to the copper analogues. While the three materials showed bactericidal activity against *E. coli*, **2-Ag** also showed strong bactericidal activity against *P. aeruginosa* (MIC 125 μg/mL), and **1-Ag** demonstrated a high sensitivity against *P. aeruginosa* (MIC 125 μg/mL and MBC 1000 μg/mL). Nevertheless, in general, in relation to the amount of metal (Ag), the materials showed better results than similar silver systems reported in the literature (MIC 5 µg/mL in reference to the amount of Ag) [47,48,49]. Nonetheless, the materials containing silver demonstrated only moderate efficacy against Gram-positive strains. The most effective MIC values were observed for the **2-Ag** material against *S. aureus*, (125 μg/mL), whereas most of the other materials had MIC values of 500 μg/mL or 1000 μg/mL. The difference in activities between Gram-positive and Gram-negative bacteria could be attributed to the fact that Gram-negative bacteria possess a thinner cell wall, which might make them more susceptible to metal ion penetration; this seems to be one of the key factors of the antibacterial mechanism of this type of material.

In addition, the antibacterial and/or bactericidal efficacy of the same materials against Gram-positive and Gram-negative clinical strains was determined (Table 4). Thus, two clinical strains of methicillin-resistant *S. aureus* (MRSA1 and MRSA2) were incubated with silver and copper materials. Subsequently, the silver materials were also incubated with two clinical strains of *P. aeruginosa* (PA8 and PA13). The MIC and MBC values of **1-Cu**, **2-Cu**, and **3-Cu**, and **1-Ag**, **2-Ag**, and **3-Ag** were determined in each case and the results are shown in Table 4.

As derived from Table 4, and with respect to the effectiveness against clinical multi-resistant *S. aureus* strains, the copper materials were shown to be inactive, while the silver analogues were shown to sensitize both clinical *S. aureus* strains (with the exception of **1-Ag** against MRSA2). In line with the observed better systematic performance of the silver-functionalized systems, these also showed high activity against clinical strains of *P. aeruginosa*. In particular, **1-Ag**, with MIC values of 62.5 μg/mL and 31.25 μg/mL, was categorized as a bacteriostatic agent against PA8 and PA13, respectively. On the other hand, **2-Ag** and **3-Ag**, with MIC values in both cases of 62.5 μg/mL and 31.25 μg/mL against PA8 and PA13, respectively, were categorized as bactericidal and bacteriostatic agents against these two clinical strains, respectively.

Therefore, silver materials were shown to inhibit the growth of *S. aureus* and *P. aeruginosa*, which are predominant in chronic wounds and burns [50,51]. The effect of phenytoin sodium encapsulation in the **1-Ag** material (yielding **1-Ag@PTN**) on wound tissue regeneration was also analyzed, since previous studies showed that silver and phenytoin sodium activities against *P. aeruginosa* [38] are additive. The MIC and MBC values of **1-Ag@PTN** against the three strains of *P. aeruginosa* were estimated and are listed in Table 5.

As can be seen in Table 5, after PTN encapsulation in the material, the MIC and MBC values decreased considerably due to the additive effect between PTN and silver when at least 2 dilutions of the material used is considered. These results were even better than those previously reported by our group for a similar system [38]. Importantly, the release of PTN in physiological medium was studied on the **1-Ag@PTN** material (see Figure S10). It was observed that after 2 h of testing in physiological medium, more than 40% of the loaded PTN had already been released into the physiological medium. 

#### 2.2.2. Minimal Biofilm Inhibitory Concentration (MBIC) and Minimal Biofilm Eradication Concentration (MBEC)

The antibiofilm effect of the silver materials was evaluated by studying the MBICs and MBECs values upon incubation of three different biofilm *P. aeruginosa* strains (ATCC 27853 and the clinical patient-derived PA8 and PA13 strains).

As can be seen in Table 6, silver materials **1-Ag**, **2-Ag**, and **3-Ag** inhibited *P. aeruginosa* biofilm growth, with the MBIC values against commercial (ATCC 27853) and clinical (PA8 and PA13) strains in the range of 250–500 μg/mL. Importantly, the silver materials were also able to exert biofilm eradication for strain PA8 at doses of 1000 μg/mL. On the other hand, and contrary to the observations in a planktonic state, an additive effect between PTN and silver was not evident in the biofilm state when comparing **1-Ag** and **1-Ag@PTN**. Thus, in most cases, the additive effect was only observed when comparing the metal content. 

#### 2.2.3. Effect on Biofilm Development

**Figure 3:**
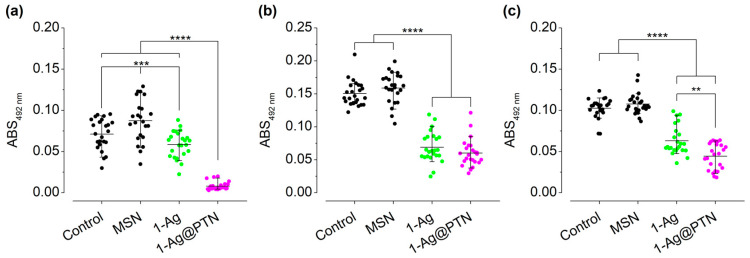
Inhibition of biofilm development of ATCC 27853 (**a**), PA8 (**b**), and PA13 (**c**) in the presence of **MSN** (as a control; in black), **1-Ag** (green), and **1-Ag@PTN** (pink) at 62.5 µg/mL. ** *p* < 0.01, *** *p* < 0.001, **** *p* < 0.0001, according to Mann–Whitney and Kruskal–Wallis tests.

The ability of **1-Ag** and **1-Ag@PTN** materials to inhibit the stages of the biofilm development of *P. aeruginosa* strains was analyzed using **MSN** material as a control (Figure 3). Following the standards [52,53,54], the study was performed using 62.5 μg/mL of each material (4 × MIC **1-Ag@PTN)**. 

As can be derived from Figure 3, the starting material (**MSN**) did not inhibit biofilm development of either collection (Figure 3a) or the clinical strains (Figure 3b,c), which was expected due to its excellent biocompatibility and the absence of any cytotoxic agent, whereas the functionalized materials did. The inhibition of ATCC 27853 strain biofilm development boosted from 16.89% with the **1-Ag** material to 91.71% with the **1-Ag@PTN** (Figure 3a). In the case of the clinical strains, both the percentages of biofilm growth inhibition and the differential performance of both types of materials were more moderate, with inhibitions of 56.02% in strain PA8 and 47.26% in strain PA13 with the **1-Ag** material, and 59.97% and 55.48% in strains PA8 and PA13, respectively, with the **1-Ag@PTN** material. Therefore, it can be concluded that the observed inhibitions on *P. aeruginosa* biofilm development is a consequence of the incorporation of silver and the differences between the two materials (most significantly on the ATCC 27853 and PA13 strains) may be due to the additive effect of silver coadministration with PTN.

#### 2.2.4. Inhibition in Wound-like Medium

**Figure 4:**
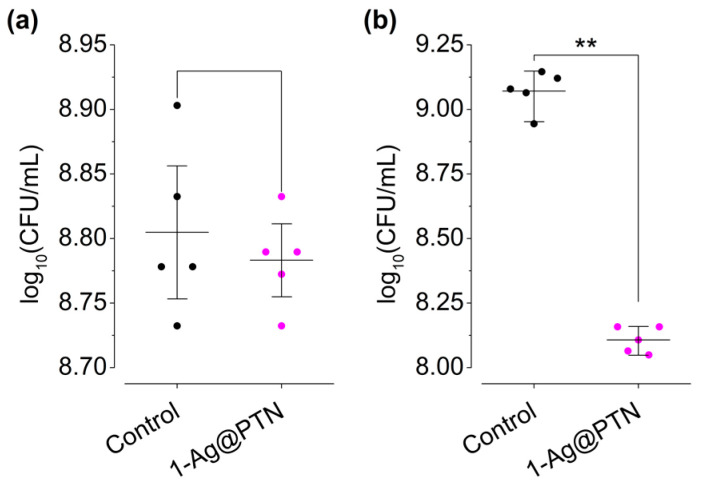
The effect of **1-Ag@PTN** against ATCC 27853 wound-like medium at 62.5 µg/mL (**a**) and 1 mg/mL (**b**). The control (black) and **1-Ag@PTN** (pink) are shown. ** *p* < 0.01 for Mann–Whitney test.

After the study in conventional culture media, an inhibition study was performed with the **1-Ag@PTN** material in a wound-like medium against *P. aeruginosa* ATCC 27853. The composition of this medium included components against which the synthesized materials would have to show their efficacy in an in vivo model. For this purpose, two concentrations of the **1-Ag@PTN** material were chosen, 62.5 μg/mL (4 × MIC) and 1 mg/mL (4 × MBC). As shown in Figure 4, the inhibition of bacterial growth was dose-dependent, showing no effect at a concentration of 62.5 μg/mL (4 × MIC) (Figure 4a), but a dramatic inhibition of 89.33% at a concentration of 1 mg/mL (Figure 4b). These results point out that the topical use of **1-Ag@PTN** at 1 mg/mL may inhibit locally pseudomonal biofilm development in wounds and burns.

#### 2.2.5. Bactericidal Mechanism of **1-Ag@PTN**

**Figure 5:**
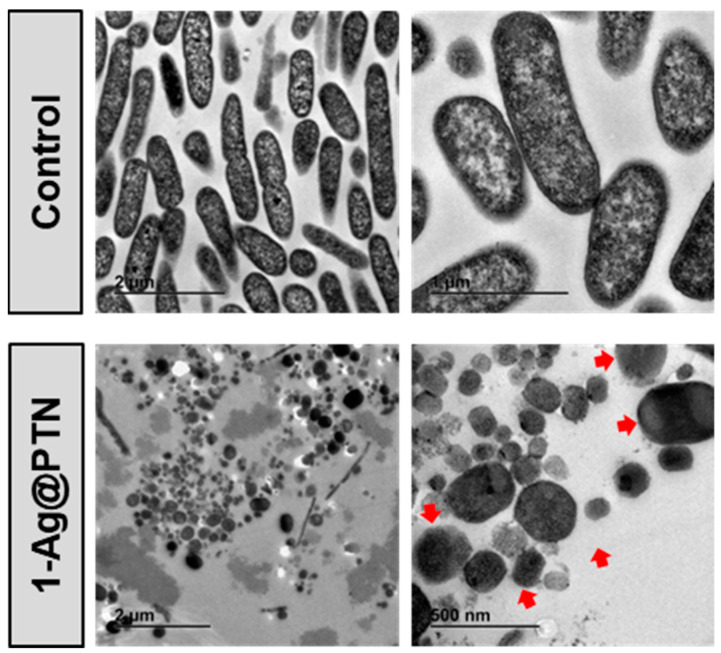
TEM images of *P. aeruginosa* ATCC 27853 treated with (top) 0 (control) and (bottom) 500 µg/mL of **1-Ag@PTN**. Red arrows indicate elements compatible with membrane vesicles.

The bactericidal mechanism of **1-Ag@PTN** was analyzed using TEM images of a *P. aeruginosa* ATCC 27853 strain culture, acquired both in the absence (control) and in the presence of 500 μg/mL (2 × MBC) of **1-Ag@PTN** (Figure 5).

As can be seen in Figure 5 (top), the control images show intact bacilli with dimensions of approximately 1.5–2 μm in length and 0.3–0.5 μm in width, whereas the TEM images acquired after incubation of the bacteria with **1-Ag@PTN** (Figure 5 bottom) showed bacteria with clear structural and morphological changes, as only elements compatible with membranous vesicles were found. Furthermore, in addition to silica nanoparticles, smaller nanoparticles that could be compatible with AgCl NPs were also observed. Silver nanoparticles have several mechanisms of action that can result in bacterial death, including interactions with the outer membrane of bacteria, interactions with intracellular components, and alterations to the respiratory chain in the cytoplasmic membrane [55,56,57]. These mechanisms could be responsible for the bactericidal effect observed in **1-Ag@PTN** against the *P. aeruginosa* ATCC 27853 strain.

#### 2.2.6. Oxidative Stress Studies and Advanced Oxidation Protein Products (AOPP)

Oxidative stress is one of the main mechanisms of bacterial toxicity of copper and silver compounds [55,58,59,60], which leads to the oxidation of essential macromolecules (particularly proteins) and induces bacterial cell death. To evaluate whether oxidative stress may be one of the potential mechanisms of the antibacterial action of **1-Cu**, **2-Cu**, and **3-Cu** ROS, advanced oxidation protein products (AOPP) were quantified upon incubation of *S. aureus* and *E. coli* with the synthesized materials at different time intervals and concentrations (MIC, MIC/10, and MIC × 10).

**Figure 6:**
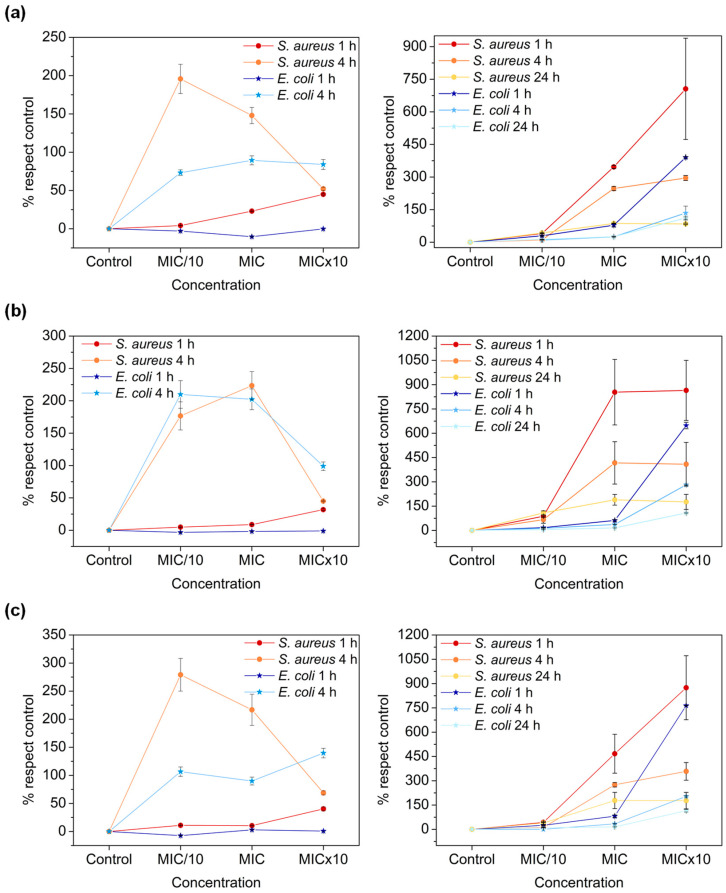
Percentage increase of reactive oxygen species at 1 and 4 h of incubation (left) and of AOPP at 1, 4, and 24 h of incubation (right) in *S. aureus* (in red, orange, and yellow) and *E. coli* (in blue) at three concentrations of the materials: (**a**) **1-Cu**, (**b**) **2-Cu**, and (**c**) **3-Cu**. The study was performed in triplicate and data are expressed as mean ± SD.

As shown in Figure 6 (left), the increase in ROS production by *E. coli* was not significant after short incubation times (1 h) and it was moderately significant (30–45%) in the case of *S. aureus* only with large concentrations of materials (MIC × 10). On the contrary, longer incubations times (4 h) at low concentrations (MIC/10) of either **1-Cu**, **2-Cu**, or **3-Cu** were enough to dramatically increase the concentration of ROS in both strains. In fact, MIC/10 was found, upon 4 h of incubation as a general rule, to be the minimum concentration to achieve maximum ROS production, which reached values as large as 279 ± 29% in *S. aureus* for **3-Cu** and 209 ± 21% in *E. coli* for **2-Cu** (see Table 7).

The generation of advanced oxidation protein products (AOPP) was observed to increase with compound concentration and decrease with time (Figure 6, right). In this sense, incubation with the MIC of **1-Cu**, **2-Cu**, or **3-Cu** was necessary to promote a significant AOPP increase in both strains, with *S. aureus* being more sensitive to treatment. On the other hand, the generation of oxidized proteins was inversely proportional to time because as the exposure time of the materials increased, the increase in AOPP was lower. This suggests that the viability of the cells decreased or the bacteria activated their proteolytic systems to restore fully oxidized proteins [61]. In addition, the AOPP levels increased with higher concentrations, with the maximum AOPP production occurring at MIC × 10. In the case of the **1-Cu** material, the levels increased to 705 ± 233% and 390 ± 4%, with respect to the control, in *S. aureus* and *E. coli*, respectively. Similar patterns were observed with the **2-Cu** and **3-Cu** materials, with increases of 864 ± 186% and 874 ± 198% for *S. aureus*, and 647 ± 20% and 764 ± 1% for *E. coli*, respectively. 

Therefore, from these results, it can be concluded that the increase of ROS and AOPP promoted by these materials in both *S. aureus* and *E. coli* could be the mechanism of bacterial death.

## 3. Materials and Methods

### 3.1. General Remarks on Characterization of the Materials 

To characterize the nanomaterials, TEM measurements were conducted using a TECNAI G2 20 TWIN instrument (FEI Company, Hillsboro, OR, USA) operating at 200 kV, equipped with a LaB6 filament and high angle annular dark-field-scanning transmission electron microscopy (HAADF-STEM). The samples were dispersed into an ethanol solvent and placed in an ultrasonic bath for 15 min before being spread onto a TEM copper grid (300 mesh) covered with a holey carbon film. The grid was then air-dried at room temperature. The textural properties of the samples were determined using N_2_ physisorption in a Tristar II Plus instrument (Micromeritics Instrument Corporation, Norcross, GA, USA). The samples were previously degassed in the SmartVacPrep system (Micromeritics Instrument Corporation, Norcross, GA, USA) at 70 °C up to a vacuum level of 1 × 10^−3^ mm Hg for 12 h and were finally analyzed at −196 °C. BET and BJH formalism of the desorption branch were used to obtain the surface area and the pore size distribution. An ICP-AES study was carried out in an Agilent 5100 Dual View. Thermogravimetric analyses (TG) were performed on a thermal analyzer TG-Q500 (TA Instruments, New Castle, DE, USA) starting from room temperature and increasing to 750 °C under a nitrogen atmosphere at a heating rate of 10 °C min^−1^. The powder XRD patterns were collected on a Phillips X’PERT powder diffractometer with CuKα radiation (λ = 1.5418 Å) in the following ranges: 0.8 < 2θ < 10° and 5 < 2θ < 90° (depending on the sample) and with a step size of 0.026°, as well as an acquisition time of 2.5 s per step at 25 °C. The Fouriertransformed infrared (IR) spectra (400–4000 cm^−1^) were recorded on a Nicolet FT-IR 6700 spectrometer in KBr pellets. The DR-UV measurements were acquired in a UV/Vis Shimadzu spectrophotometer. These spectra were recorded at room temperature with BaSO_4_ as a reference material. ^13^C CP MAS NMR solid-state NMR measurements were carried out on the powder samples and recorded in a high-resolution mode, at 298 K, on a Bruker Avance 400 WB spectrometer (Bruker, MA, USA) at 9.4 T, using 100.66 and 400.17 MHz resonance frequencies (^1^H and ^13^C, respectively). The ^13^C experiments were performed with cross-polarization (CP), high-power decoupling, and magic angle spinning (MAS), using a Bruker double-bearing probe head (Bruker, MA, USA) and 4 mm zirconia rotors driven by dry air. The MAS rates were 10 kHz. The Hartmann–Hahn conditions for ^13^C were matched using adamantane. The recycle delay was 5 s and the contact time was 2 ms. The chemical shifts used glycine (Gly) as an external standard (dCO of Gly = 176.5 ppm). 

The quantification of the absorbance in the in vitro studies was carried out using the Tecan Infinite M200 Plate Reader (Tecan, Switzerland). The absorbances used were 492 nm using 620 nm as a reference filter, 570 nm, and 600 nm. The bactericidal mechanism of the materials was observed using a Jeol JEM 1400 transmission electron microscope (Jeol Ltd., Tokyo, Japan). For this purpose, sections were collected on 200 mesh nickel grids. The Ultracut UC7 ultramicrotome (Leica Microsystems, Wetzlar, Germany) was used for sample preparation.

### 3.2. Synthesis of Mesoporous Silica Nanoparticles (MSNs) 

MSN nanoparticles were synthesized by a modification of the method previously published by Zhao and coworkers [62]. In summary, an aqueous solution of 2.74 mmol of CTAB (N-ethyltrimethylammonium bromide) was dissolved in 480 mL of water. Sodium hydroxide (2.0 M, 3.5 mL) was added at room temperature and the mixture was heated up to 80 °C. Then, 5 mL (22.4 mmol) of the silica precursor TEOS (tetraethyl orthosilicate) was added dropwise and vigorously stirred for 2 h. The white precipitate was isolated by filtration, washed with abundant water and methanol, and dried for 24 h at 80 °C in an oven. Finally, a calcination process was carried out at 550 °C for 24 h with a temperature ramp of 1 °C/min.

### 3.3. Functionalization of Silica Materials with Amino Ligand: Synthesis of *****MSN–AP*****

To functionalize the MSN with 3-aminopropyltriethoxysilane, a mass ratio of 1:2 MSN to AP was employed. The MSN was first dehydrated by subjecting it to vacuum treatment at 80 °C for 24 h. The dehydrated MSN was then suspended in 50 mL of dry toluene, followed by addition of the amino ligand to the mixture, which was stirred at 110 °C for 48 h. After this period, the resulting suspension was centrifuged at 6000 rpm for 10 min, and the solid fraction was washed thoroughly with toluene and diethyl ether. The resulting white powder was then dried at 80 °C on a stove for 24 h. The material obtained was named **MSN–AP**.

### 3.4. Preparation of ***MSN–AP*** with Fluoroquinolone Ligand: Synthesis of Materials 1, 2, and 3

The fluoroquinolones used were 1-cyclopropyl-6,7,8-trifluoro-4-oxo-1,4-dihydroquinolone-3-carboxylic acid (3F-FQ), 1-cyclopropyl-6,7-difluoro-4-oxo-1,4-dihydroquinolone-3-carboxylic acid (2F-FQ), and 7-chloro-1-cyclopropyl-6-fluoro-4-oxo-1,4-dihydroquinolone-3-carboxylic acid (3F-FQ).

The theoretical level of 10% w/w SiO_2_/FQ was used to functionalize the **MSN–AP**. To incorporate the fluoroquinolone ligand, an EDAC coupling process was carried out in an MES buffer. Initially, a DMSO solution containing the corresponding fluoroquinolone ligand was added to the MES buffer, along with EDAC and NHS, in a molar proportion of 1:2.5. The resulting mixture was subjected to vigorous stirring for 15 min at room temperature. After this, **MSN–AP** was added, and the resulting solution was stirred for an additional 2 h at room temperature. Finally, the solid was isolated by centrifugation (6000 rpm, 10 min) and washed with DMSO, water, ethanol, and diethyl ether to produce materials **1** (MSN-AP-3F-FQ), **2** (MSN-AP-2F-FQ), and **3** (MSN-AP-FCl-FQ).

### 3.5. Preparation of Copper and Silver Materials

For the synthesis of the copper-containing materials, a suspension of the materials previously functionalized with fluoroquinolone ligand, namely, **1** (MSN-AP-3F-FQ), **2** (MSN-AP-2F-FQ), and **3** (MSN-AP-FCl-FQ), was stirred in 60 mL of acetonitrile. With the aim of preparing materials with a 1:1 molar ratio (ligand/copper), a composition of 10% by mass of the ligand was assumed in each case, and the appropriate amount of Cu(NO_3_)_2_·2.5H_2_O was added, followed by triethylamine (Et_3_N) in a 1:1 base:Cu molar ratio. The reaction was refluxed for 24 h. The product was isolated by centrifugation (6000 rpm, 10 min) and washed with acetonitrile (×3) and water (×3). The materials were named **1-Cu**, **2-Cu**, and **3-Cu**.

The silver materials were synthesized using the same procedure as described for the copper materials. In this case, the metal salt used was AgNO_3_ and the reaction was kept in the dark. The synthesized materials were named **1-Ag**, **2-Ag**, and **3-Ag**.

### 3.6. Preparation of ***1-Ag@PTN*** Material

In order to load the **1-Ag** material with phenytoin sodium (PTN), a 1 mL saturated solution of the PTN in water was added to 10 mg of the **1-Ag** material. This mixture was kept at 500 rpm and 4 °C for 20 h. Subsequently, the solid product was isolated by centrifugation (6000 rpm, 10 min) and washed once with water and ethanol in a ratio of 1:1. The resulting product was dried at room temperature. The phenytoin sodium-loaded material was named **1-Ag@PTN**.

### 3.7. In Vitro Studies

#### 3.7.1. Bacteria

Four types of bacteria from the American Type Culture Collection (ATCC) were studied: two of them were gram-negative (*Escherichia coli* ATCC 25922 and *Pseudomonas aeruginosa* ATCC 27853) and another two were gram-positive (*Staphylococcus aureus* ATCC 29213 and *Enterococcus faecalis* ATCC 29212). In addition to these bacteria, two clinical strains of *P. aeruginosa*, PA8 and PA13, and two clinical strains of methicillin-resistant *S. aureus* (MRSA), MRSA1 and MRSA2, isolated in the Clinical Microbiology department of the Fundación Jimenez Díaz, were used (Table 8). All the strains were stored frozen at −80 °C until the experiments were performed.

#### 3.7.2. Minimum Inhibitory Concentration and Minimum Bactericidal Concentration

The standardized Clinical and Laboratory Standards Institute’s microdilution method was used to determine the minimum inhibitory concentrations (MIC) of the seven materials [63]. The inoculum was prepared by diluting the cultures overnight to a cell concentration of 1–5 × 10^8^ colony-forming unit per mL (CFU/mL), corresponding to 0.5 on the McFarland scale, which was then diluted 1:100 in Mueller Hinton Broth (MHB) medium. Serial dilutions were performed from 2000 to 1.953 µg/mL in a 96-well microplate, and the inoculum was added at a concentration of 1–5 × 10^6^ CFU/mL. After incubating for 18 h at 37 °C and 5% CO_2_, MTT (3-(4,5-dimethyl-2-thiazolyl)-2,5-diphenyltetrazole bromide) was used as the developing agent to determine the lowest concentration where no microbial growth was observed after 18 h of incubation, which was defined as the MIC.

The minimum bactericidal concentration (MBC) was determined using the flash microdilution method with some modifications [64]. Briefly, after 24 h of incubation, 20 μL of each well was mixed with 180 μL of tryptic soy broth (TSB) in a new 96-well plate and incubated at 37 °C and 5% CO_2_ for 24 h. The absorbance at 600 nm was measured to determine the bactericidal concentration of each well, and the MBC was defined as the lowest concentration where the death of 99.9% of the initial inoculum occurred.

#### 3.7.3. Minimal Biofilm Inhibitory Concentration and Minimal Biofilm Eradication Concentration

The minimum inhibitory concentrations (MBIC) and minimum biofilm eradication concentrations (MBEC) were determined using the methodology previously described [65]. The MBIC is the minimum concentration necessary to inhibit the visible growth of a bacterial biofilm. To determine the MBIC, biofilm formation was induced on the pegs of the Calgary device. For this purpose, a 96-well plate (Thermo Fisher Scientific, Waltham, MA, USA) was inoculated with 200 µL of TSB containing 10^6^ CFU/mL bacteria per well. The lid (Thermo Fisher Scientific, Waltham, MA, USA) of the Calgary device was then put in place, and the plate was incubated at 37 °C and 5% CO_2_ for 24 h. After incubation, the lid pegs were rinsed twice using wells containing 200 µL of 0.9% NaCl saline. The lid was then placed on a plate with different concentrations of the tested materials, ranging from 1000 to 1.953 µg/mL, with a twofold dilution; MHB was added to a final volume of 200 µL per well. This plate was incubated at 37 °C and 5% CO_2_ for at least 20 h. After incubation, the MBIC values were determined using MTT as the developing agent. For this purpose, 20 µL of MTT was added to each well and shaking was induced at 90 rpm in an incubator at 37 °C for 1.5 h. After incubation, the absorbance was measured at 570 nm. The experiments were performed in triplicate.

MBEC is the minimum concentration necessary to kill a bacterial biofilm. For MBEC, the MBIC lid was rinsed twice in a plate with wells containing 200 µL of 0.9% NaCl saline, placed in a plate with 200 µL of TSB, and statically incubated at 37 °C and 5% CO_2_ for 24 h. After incubation, the MBEC was determined by measuring the absorbance at 600 nm.

#### 3.7.4. Effect on Biofilm Development 

To study whether the materials inhibit biofilm development, one 96-well plate was used for each *P. aeruginosa* strain. The materials used were unmodified **MSN** as a control and **1-Ag** loaded and unloaded with phenytoin sodium (**1-Ag@PTN** and **1-Ag**). The concentration used for each material was 4 times the MIC of the drug-containing material, considering previous studies that state that 4 × MIC is a good therapeutic approach [52,53,54]. For this purpose, 200 µL of 1 × 10^6^ CFU/mL in TSB + 1% glucose of each strain with a 4 × MIC concentration of the material was deposited in a 96-well flat-bottom plate and incubated at 37 °C and 5% CO_2_ for 24 h. After incubation, each well was rinsed twice with 200 µL of saline. Biofilm quantification was performed according to a previously reported methodology [66]. To begin with, each well was fixed with 200 µL of MeOH and kept for 20 min in open air. The supernatant was then removed and allowed to dry at 60 °C. After fixation, staining was performed; thus, 150 µL of 1% safranin was added for 15 min to allow the dye to penetrate through the biofilm. Finally, each well was rinsed twice with 200 µL of distilled water and then was solubilized with absolute ethanol. Finally, the absorbance was measured at 492 nm in a colorimeter using 620 nm as a reference filter. This experiment was performed in eight wells for each material and strain and in triplicate (*n* = 24).

#### 3.7.5. Inhibition in Wound-like Medium

Biofilm development in a wound-like medium was determined using previously described methods based on the Lubbock chronic wound medium [67,68]. The wound-like medium was composed of 50% bovine plasma (Sigma-Aldrich), 45% Bolton broth (Sigma-Aldrich, St. Louis, MO, USA), and 5% lacquered horse red blood cells (Thermo Fisher Scientific, Waltham, MA, USA), supplemented with or without (positive control) the tested material at a concentration of 4 times MIC or 4 times MBC. Half a milliliter of each medium was incubated with 5 µL of 10^8^ CFU/mL of *P. aeruginosa* strain ATCC 27853 for 24 h at 37 °C and 5% CO_2_. After incubation, 0.5 mL of saline was added and sonicated for 5 min. Then, this sonicated saline was serially diluted with saline, and the CFU per milliliter was estimated by the drop plate method on MacConkey agar plates. This experiment was performed in quintuplicate for each material (*n* = 5).

#### 3.7.6. Bactericidal Mechanism of the **1-Ag@PTN** Material Using TEM

The bactericidal mechanism of the final **1-Ag@PTN** material in strain ATCC 27853 was analyzed by transmission electron microscopy (TEM). The concentration of the material used was 2 × MBC. The protocol used for this was described previously [69]. Thin sections (60 nm) were cut for TEM of the resin-included bacteria using a Leica Ultracut ultramicrotome UC7 (Leica Microsystems, Germany). Sections were collected on 200-mesh nickel grids and examined with a Jeol JEM 1400 transmission mission electron microscope (Jeol Ltd., Tokyo, Japan).

#### 3.7.7. Oxidative Stress Studies

Bacterial suspensions (100 μL) of *S. aureus* ATCC 29213 and *E. coli* ATCC 25922 were incubated with 100 μL of each copper material (**1-Cu**, **2-Cu**, and **3-Cu**) using phosphate buffered saline (PBS) as a control and sub-MIC (MIC/10), MIC, and supra-MIC (MIC × 10) concentrations of each material for 1 and 4 h at 37 °C. After incubation, 20 μL of a 20 μM aqueous solution of 2′,7′-dichlorodihydrofluorescein diacetate diacetoxymethyl ester (H2-DCFDA, Sigma-Aldrich) was added. After 30 min, the fluorescence intensity was measured on a Biotek Synergy HT spectrofluorometer (BioTek Instruments, Winooski, VT, USA), with excitation wavelengths of 480 nm and emission wavelengths of 520 nm. Non-treated bacterial suspensions were used as controls. The experiments were carried out in triplicate (*n* = 3).

#### 3.7.8. Advanced Oxidation Protein Products (AOPP)

The AOPP levels were determined by the spectrophotometric method, using chloramine T as a reference, which, in the presence of KI, absorbs at 340 nm. Overnight cultures in TSB of *S. aureus* ATCC 29213 and *E. coli* ATCC 25922 were placed in contact with 0.5 mL suspensions of the studied materials (**1-Cu**, **2-Cu**, and **3-Cu**) at corresponding sub-MIC (MIC/10), MIC, and supra-MIC (MIC × 10) concentrations for 1, 4, and 24 h. PBS (pH 7) was used as a control. After the incubation periods, to 100 μL of each sample, a mixture of 50 μL of 1.16 M KI and 50 μL of acetic acid were added and the absorbance was measured at 340 nm with the Sunrise microplate reader (Tecan Trading AG, Switzerland). The experiments were performed in triplicate (*n* = 3).

#### 3.7.9. PTN Release Studies

To evaluate the stability of the **1-Ag@PTN** material and the amount of PTN released at different times, a PTN release experiment was conducted. Approximately 1 mg of the **1-Ag@PTN** material was dispersed in a mixture of 4 mL 95:5 PBS buffer (pH = 7.4):DMSO, and incubated with slight shaking (30 rpm) at 37 °C for varying durations using a Roto-587 Therm incubator (Benchmark Scientific, Sayreville, NJ, USA) with temperature control. The resulting suspensions were filtered through a 0.2 µm nylon filter and analyzed by HPLC (Flexar Perkin-Elmer, Waltham, MA, USA) using a C_18_ column (COSMOSIL 5C_18_-MS-II 4.6 mm I.D. × 250 mm with a particle size of 4.4 µm) and a mobile phase consisting of 60:40 MeOH:H_2_O at a flow rate of 0.7 mL/min. The PTN was detected in the chromatograms at a retention time of 9.2 min.

### 3.8. Statistical Analysis

GraphPad Prism 8 software (version 8.01) was used to conduct statistical analyses. To compare two groups, a two-sided Mann–Whitney nonparametric test was employed, while a Kruskal–Wallis nonparametric test was utilized for comparing more than two groups. The statistical significance was set at *p*-values ≤ 0.05, and all the results were expressed as the median and interquartile range.

## 4. Conclusions

In this study, seven materials based on mesoporous silica nanoparticles functionalized with halogenated derivatives of FQ with Ag^+^ or Cu^2+^ were synthesized and characterized. The evaluation of MIC and MBC was performed against two Gram-positive strains (*S. aureus* and *E. faecalis*) and two Gram-negative strains (*E. coli* and *P. aeruginosa*). The Cu^2+^ systems only showed antibacterial activity against *S. aureus* and *E. coli*, and, according to the oxidative stress study, this activity could mainly be due to the increase in ROS generation, as they can increase the generation of this type of reactive species by almost three times. However, although the Cu^2+^ materials showed bactericidal activity against *E. coli* and *S. aureus*, they were not active against clinical MRSA strains. Nevertheless, the Ag^+^ materials showed a wider spectrum of activity, and were even able to inhibit clinical strains of MRSA and *P. aeruginosa*, which are usually less susceptible to or more resistant to antibiotics.

On the other hand, all the silver materials inhibited biofilm growth, although eradication was only successful in the case of the PA8 strain. Furthermore, in the search for a bifunctional material capable of inhibiting bacterial biofilm and promoting tissue regeneration, the proliferative drug sodium phenytoin was encapsulated in the **1-Ag** material, resulting in the material named **1-Ag@PTN**. After encapsulation, the MIC and MBC values of the loaded material were determined, and it was observed that the concentrations decreased significantly due to the additive effect between silver and PTN. However, in the biofilm state, the minimum concentrations did not undergo significant changes; moreover, the silver materials showed no capacity to eradicate the biofilm. Despite this, the inhibitory capacity of **1-Ag** and **1-Ag@PTN** materials to prevent biofilm development was evaluated, and once again, the additive effect caused by the co-administration of silver with sodium phenytoin was observed, since in the ATCC 27853 and PA13 strains, the loaded material showed greater reduction in development.

Finally, the **1-Ag@PTN** material showed, in the worst case, the ability to reduce biofilm development by up to 55% and inhibit bacterial growth in a “wound-like medium” by up to 89.33%, making it a promising material that should continue to be explored for the treatment of chronic wound infections with further studies in vivo.

In general, the materials reported here have antibacterial activities comparable to other systems based on recent approaches that have used nanocomposites, such as Ag_2_S quantum dots [70], biopolymer hydrogels reinforced by copper/tannic acid nanosheets [71], or enzyme-silver-polymers nanocomposites [72]. In this context, our current nanosystems seem to need a comparable, if not somewhat lower amount of copper or silver; however, they do not require other activating sources (light or oxidative species) to demonstrate similar activities. In this context, the nanosystems reported here may be beneficial to superficial wound infections when appropriately formulated for topical use. This will be one of the main objectives of our research teams in future studies.

## Figures and Tables

**Table 1:** Textural parameters obtained by nitrogen adsorption/desorption isotherms.

Material	BET Surface (m^2^/g)	Pore Volume (cm^3^/g)	Pore Diameter (nm)
**MSN**	925	0.77	2.72
**1-Cu**	45	0.09	<2.00
**1-Ag**	42	0.09	<2.00
**1-Ag@PTN**	27	0.07	<2.00
**2-Cu**	42	0.10	<2.00
**2-Ag**	39	0.11	<2.00
**3-Cu**	144	0.12	<2.00
**3-Ag**	39	0.10	<2.00

**Table 2:** Quantification of ligands, metals, and PTN by TG or ICP-AES. Values are given in mmol per gram of material.

Material	AP	FQ	Cu	Ag	PTN
**1-Cu**	0.70	0.28	0.20	-	-
**1-Ag**	0.70	0.28	-	0.32	-
**1-Ag@PTN**	0.70	0.28	-	0.23	1.16
**2-Cu**	0.70	0.29	0.15	-	-
**2-Ag**	0.70	0.29	-	0.13	-
**3-Cu**	0.70	0.36	0.13	-	-
**3-Ag**	0.70	0.36	-	0.10	-

**Table 3:** MIC and MBC values of the final functionalized materials against commercial bacterial strains in planktonic state. Values are given in μg of material/mL and μg metal/mL (in brackets).

Material	*S. aureus* ATCC 29213		*E. faecalis* ATCC 29212	
	MIC	MBC	MIC	MBC
**MSN**	500	1000	500	1000
**1-Cu**	250 (3.25)	250 (3.25)	>2000 (26.00)	>2000 (26.00)
**2-Cu**	500 (4.50)	500 (4.50)	>2000 (18.00)	>2000 (18.00)
**3-Cu**	250 (2.00)	500 (4.00)	>2000 (16.00)	>2000 (16.00)
**1-Ag**	500 (17.50)	>2000 (70.00)	1000 (35.00)	>2000 (70.00)
**2-Ag**	125 (1.88)	>2000 (30.00)	500 (7.50)	>2000 (30.00)
**3-Ag**	1000 (10.00)	>2000 (20.00)	1000 (10.00)	>2000 (20.00)
**Material**	***E. coli* ATCC 25922**		***P. aeruginosa* ATCC 27853**	
	**MIC**	**MBC**	**MIC**	**MBC**
**MSN**	250	1000	250	500
**1-Cu**	15.62 (0.20)	15.62 (0.20)	>2000 (26.00)	>2000 (26.00)
**2-Cu**	15.62 (0.14)	15.62 (0.14)	>2000 (18.00)	>2000 (18.00)
**3-Cu**	15.62 (0.12)	31.25 (0.25)	>2000 (16.00)	>2000 (16.00)
**1-Ag**	250 (8.75)	500 (17.50)	125 (4.38)	1000 (35.00)
**2-Ag**	250 (3.75)	250 (3.75)	125 (1.88)	250 (3.75)
**3-Ag**	250 (2.50)	500 (5.00)	250 (2.50)	2000 (20.00)

**Table 4:** MIC and MBC values of the final functionalized materials against two clinical multi-resistant strains of *S. aureus* (MRSA1 and MRSA2) and two clinical strains of *P. aeruginosa* (PA8 and PA13) in a planktonic state. Values in μg of material/mL and, in brackets, in μg metal/mL.

Material	*S. aureus* MRSA1		*S. aureus* MRSA2	
	MIC	MBC	MIC	MBC
**1-Cu**	>2000 (26.00)	>2000 (26.00)	>2000 (26.00)	>2000 (26.00)
**2-Cu**	>2000 (18.00)	>2000 (18.00)	>2000 (18.00)	>2000 (18.00)
**3-Cu**	>2000 (16.00)	>2000 (16.00)	>2000 (16.00)	>2000 (16.00)
**1-Ag**	1000 (35.00)	2000 (70.00)	2000 (70.00)	>2000 (70.00)
**2-Ag**	1000 (15.00)	2000 (30.00)	1000 (15.00)	>2000 (30.00)
**3-Ag**	1000 (10.00)	2000 (20.00)	1000 (10.00)	2000 (20.00)
**Material**	***P. aeruginosa* PA8**		***P. aeruginosa* PA13**	
	**MIC**	**MBC**	**MIC**	**MBC**
**1-Ag**	62.5 (2.19)	250 (8.75)	31.25 (1.09)	500 (17.50)
**2-Ag**	62.5 (0.94)	125 (1.88)	31.25 (0.47)	500 (7.50)
**3-Ag**	62.5 (0.63)	125 (1.25)	31.25 (0.31)	500 (5.00)

**Table 5:** MIC and MBC values of **1-Ag@PTN** against three different *P. aeruginosa* strains in a planktonic state. Data are given in µg/mL of material, and data in brackets refer to the µg/mL of silver.

Material	ATCC 27853		PA8		*P. aeruginosa* PA13	
	MIC	MBC	MIC	MBC	MIC	MBC
**1-Ag@PTN**	15.625 (0.39)	250 (6.25)	15.625 (0.39)	125 (3.13)	15.625 (0.39)	250 (6.25)

**Table 6:** MBIC and MBEC values for silver materials against three different *P. aeruginosa* strains in biofilm state. Data are given in µg/mL of material, and data in brackets refer to the µg/mL of silver.

Material	ATCC 27853		PA8		PA13	
	MBIC	MBEC	MBIC	MBEC	MBIC	MBEC
**1-Ag**	250 (8.75)	>1000 (35.00)	500 (17.50)	1000 (35.00)	500 (17.50)	>1000 (35.00)
**1-Ag@PTN**	250 (6.25)	>1000 (25.00)	250 (6.25)	1000 (25.00)	1000 (25.00)	>1000 (25.00)
**2-Ag**	250 (3.75)	>1000 (15.00)	500 (7.50)	1000 (15.00)	500 (7.50)	>1000 (15.00)
**3-Ag**	500 (5.00)	>1000 (10.00)	500 (5.00)	1000 (10.00)	500 (5.00)	>1000 (10.00)

**Table 7:** Maximum ROS and AOPP production increases (%) obtained and the concentrations and incubation times required.

*S. aureus*						
Material	ROS			AOPP		
	Concentration	t (h)	%	Concentration	t (h)	%
**1-Cu**	MIC/10	4	195 ± 19	MIC × 10	1	705 ± 233
**2-Cu**	MIC	4	223 ± 22	MIC × 10	1	864 ± 186
**3-Cu**	MIC/10	4	279 ± 29	MIC × 10	1	874 ± 198
***E. coli***						
**Material**	**ROS**			**AOPP**		
	**Concentration**	**t (h)**	**%**	**Concentration**	**t (h)**	**%**
**1-Cu**	MIC	4	89 ± 6	MIC × 10	1	390 ± 4
**2-Cu**	MIC/10	4	209 ± 21	MIC × 10	1	647 ± 20
**3-Cu**	MIC × 10	4	139 ± 8	MIC × 10	1	764 ± 1

**Table 8:** Antibiotic susceptibility pattern of the clinical strains of *S. aureus* and *P. aeruginosa* studied in this work. Data are given in mg/L. S: susceptible; R: resistant; Pos: positive; Neg: negative.

Antibiotic	*S. aureus*		*P. aeruginosa*	
	MRSA1	MRSA2	PA8	PA13
Amikacin	-	-	S (2)	S (2)
Aztreonam	-	-	S (4)	S (2)
Cefepime	-	-	S (1)	R (16)
Cefoxitin detection	Pos	Pos	-	-
Ceftazidime	-	-	S (2)	S (8)
Ceftolozane/Tazobactam	-	-	S (0.5)	S (1)
Ciprofloxacin	-	-	S (≤0.25)	R (≥4)
Clindamycin	S (≤0.25)	R (≥8)	-	-
Colistin	-	-	S (≤0.5)	S (≤0.5)
Cotrimoxazole	S (≤10)	S (20)	-	-
Daptomycin	S (0.25)	S (0.25)	-	-
Erythromycin	R (≥8)	R (≥8)	-	-
Fosfomycin	S (≤8)	S (32)	-	-
Fusidic Acid	S (≤0.5)	S (≤0.5)	-	-
Gentamycin	S (≤0.5)	R (≥16)	S (≤1)	S(≤1)
Imipenem	-	-	S (1)	S (1)
Inducible clindamycin resistance	Neg (≥8)	Neg	-	-
Levofloxacin	R (≥8)	R (≥8)	-	-
Linezolid	S (2)	S (2)	-	-
Meropenem	-	-	-	-
Mupirocin	-	R (≥512)	S (≤0.25)	S (1)
Oxacillin	R (≥4)	R (≥4)	-	-
Penicillin	R (≥0.5)	R (≥0.5)	-	-
Piperacillin-tazobactam	-	-	-	-
Teicoplanin	S (≤0.5)	S (≤0.5)	S (≤4)	R (≥128)
Tobramycin	R (≥16)	R (≥16)	-	-
Vancomycin	S (1)	S (≤0.5)	S (≤1)	S (≤1)

## Data Availability

Data is contained within the article and Supplementary Materials.

## Supplementary Materials

The following supporting information can be downloaded at: https://www.mdpi.com/article/10.3390/ph16070961/s1, Figure S1: Particle size distributions of (a) **1-Cu**, (b) **1-Ag**, (c) **1-Ag@PTN**, (d) **2-Cu**, (e) **2-Ag**, (f) **3-Cu**, and (g) **3-Ag**; Figure S2: Nitrogen adsorption (solid line) and desorption (dashed line) isotherms of materials based on (a) **1**, (b) **2**, and (c) **3**; Figure S3: Pore size distribution of materials based on **1** (a), **2** (b), and **3** (c); Figure S4: Thermogravimetric studies of materials based on **1** (a), **2** (b), and **3** (c); Figure S5: Small-angle XRD patterns of materials based on **2** (a) and **3** (b); Figure S6: XRD diffractograms of (a) **1-Ag**, **2-Ag**, and **3-Ag** with AgCl and (b) **1-Ag@PTN** with MSN@PTN; Figure S7: FT-IR spectra of the starting material MSN and final functionalized materials based on (a) **1**, (b) **2**, and (c) **3**; Figure S8: DR-UV spectra of the final materials based on (a) **1**, (b) **2**, and (c) **3**; Figure S9: 13C CP MAS NMR spectra of materials (a) **1**, (b) **2**, and (c) **3**; Figure S10: PTN release in **1-Ag@PTN**; Table S1: XRD data of MSN and its functionalized materials.
