# Supplementary material for: Antibacterial Properties of Mesoporous Silica Nanoparticles Modified with Fluoroquinolones and Copper or Silver Species
Source: Pharmaceuticals (Basel). 2023 Jul 5;16(7):961. doi: 10.3390/ph16070961 (PMC10386262; doi:10.3390/ph16070961)
Supplement: Supplementary file 1 [file pharmaceuticals-16-00961-s001.zip › pharmaceuticals-2446869-supplementary.pdf]

SUPPORTING INFORMATION

Antibacterial Properties of Mesoporous Silica Nanoparticles modified with Fluoroquinolones and Copper or Silver Species

1.1. *Physicochemical characterization of functionalized NPs.*

1. Particle size distributions of final materials
2. Textural properties. Nitrogen adsorption-desorption isotherms and pore size distribution of MSN and the final materials
3. TG of the functionalized materials.
4. Characterization by powder X-ray diffraction studies.
5. FTIR and UV-Vis spectroscopy
6. Solid State RMN

1.2. *In Vitro Studies of Antibacterial Activity*

1. PTN release studies

1.1. Physicochemical characterization of functionalized NPs.

1.1.1. Particle size distribution of final materials

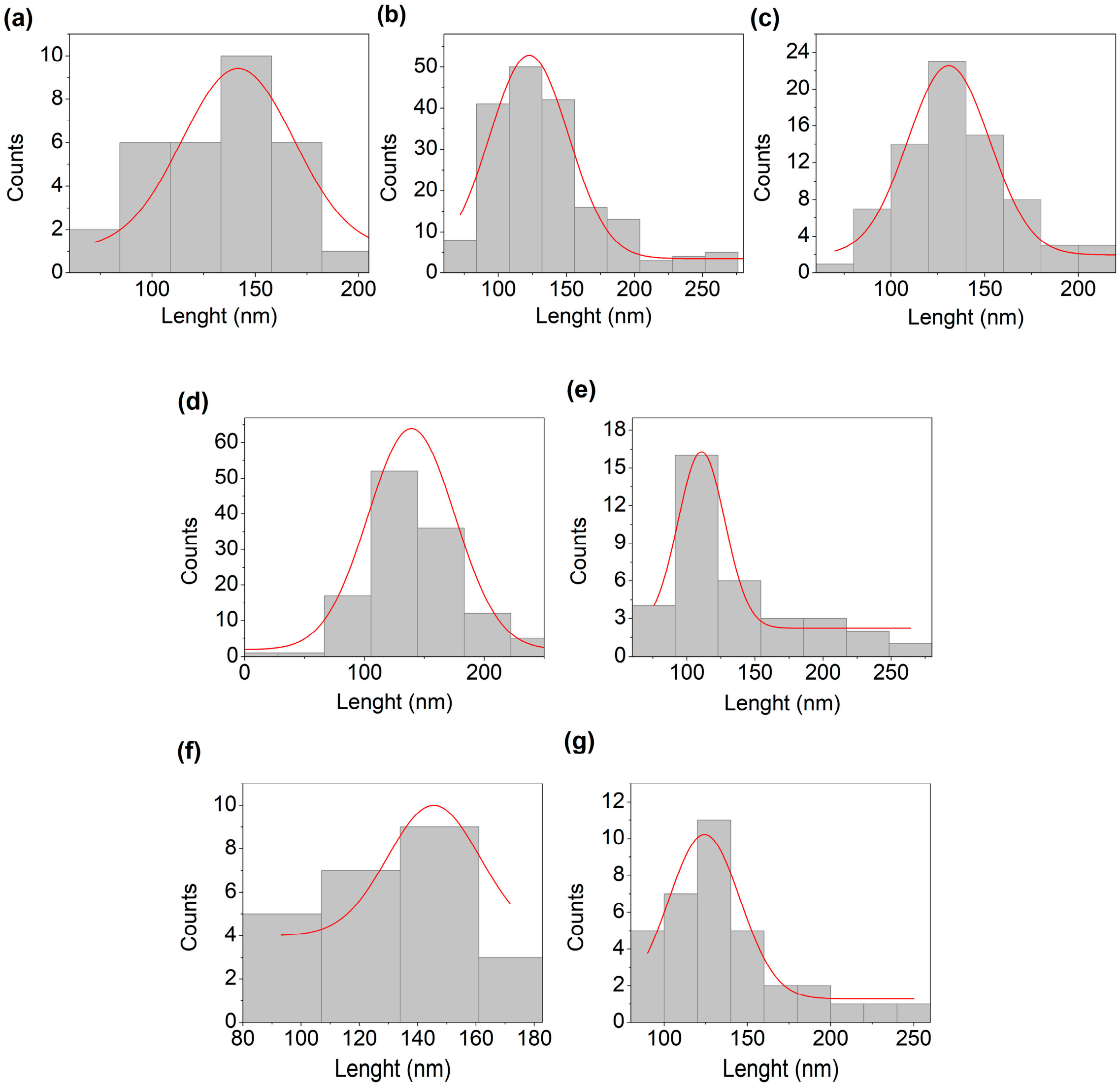

Figure S1. Particle size distributions of (a) **1-Cu**, (b) **1-Ag**, (c) **1-Ag@PTN**, (d) **2-Cu**, (e) **2-Ag**, (f) **3-Cu** and (g) **3-Ag**.

1.1.2. Nitrogen adsorption-desorption isotherms and pore size distribution

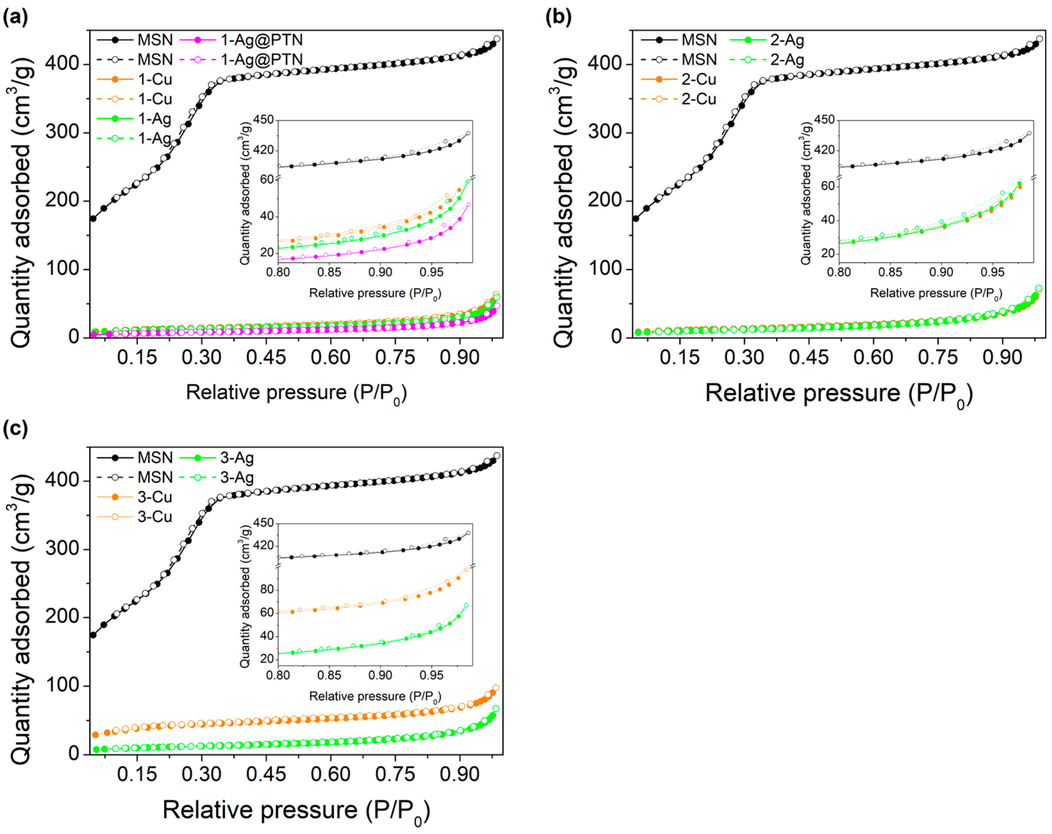

Figure S2. Nitrogen adsorption (solid line) and desorption (dashed line) isotherms of materials based on (a) **1**, (b) **2** and (c) **3**.

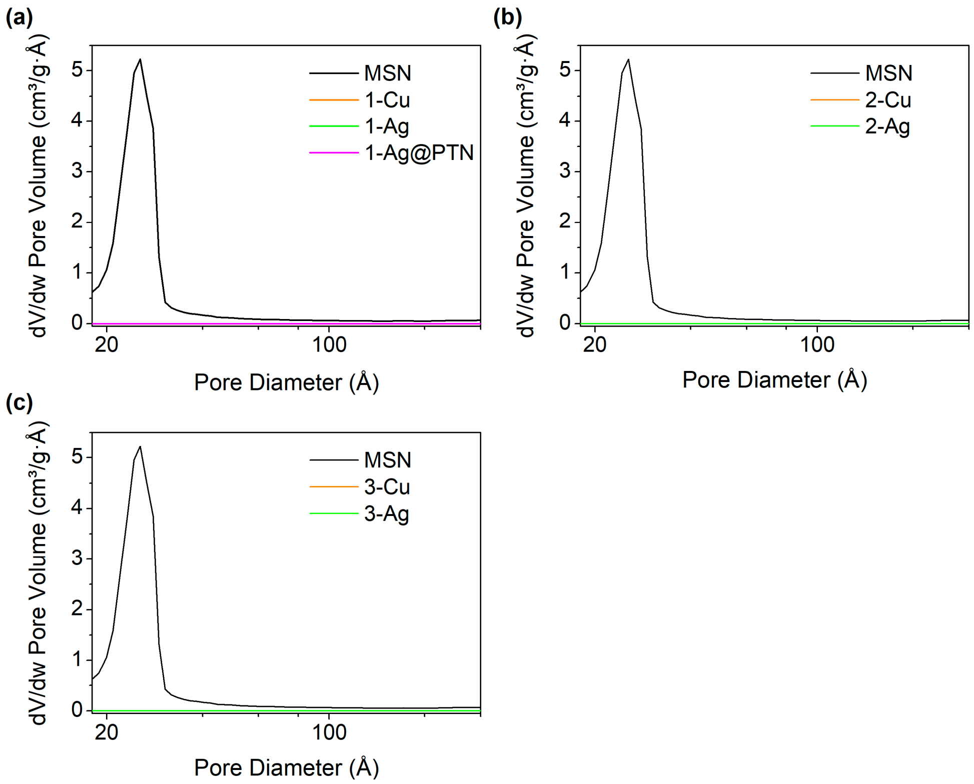

Figure S3. Pore size distribution of materials based on **1** (a), **2** (b) and **3** (c).

1.1.3. TG of the functionalized materials

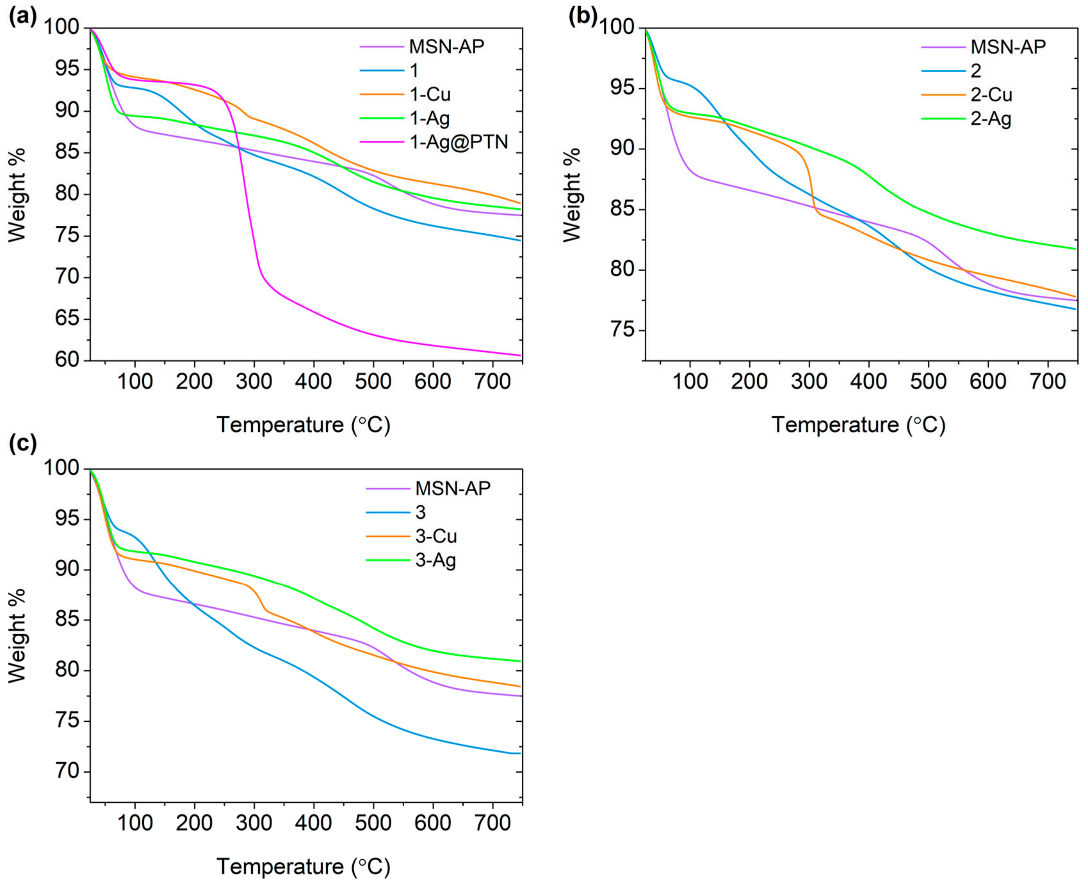

Figure S4. Thermogravimetric studies of materials based on **1** (a), **2** (b) and **3** (c).

1.1.4. Powder X-ray diffraction

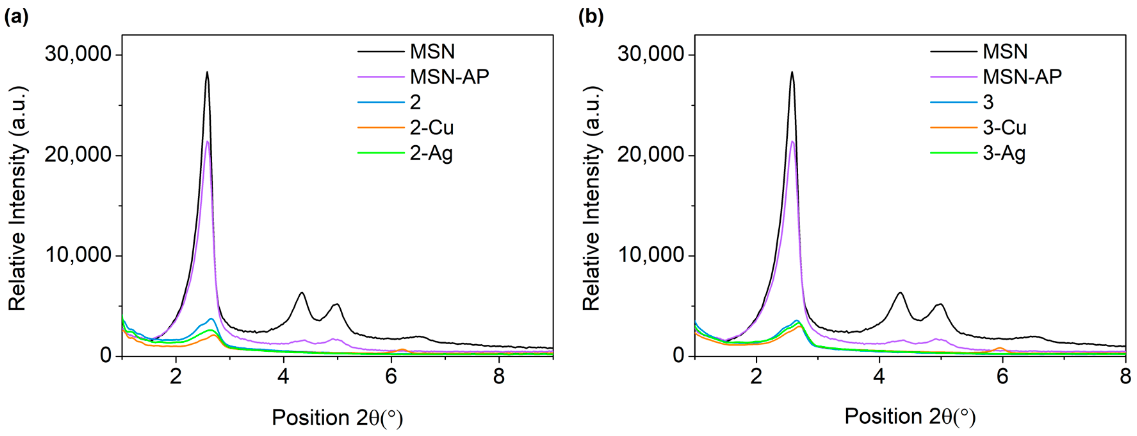

Figure S5. Small-angle XRD patterns of materials based on **2** (a) and **3** (b).

Table S1. XRD data of MSN and its functionalized materials.

Material	*hkl*	2θ(°)	d_{hkl} (nm)	a_0 (nm)
	100	2.57	3.44	3.97
**MSN**	110	4.32	2.04	2.36
	200	4.98	1.77	2.05
**MSN-AP**	100	2.50	35.32	40.78
**1**	100	100	2.58	3.42
**1-Cu**	100	100	2.63	3.35
**1-Ag**	100	100	2.66	3.32
**1-Ag@PTN**	100	100	2.63	3.35
**2**	100	100	2.63	3.35
**2-Cu**	100	100	2.66	3.32
**2-Ag**	100	100	2.71	3.26
**3**	100	100	2.63	3.35
**3-Cu**	100	100	2.66	3.32
**3-Ag**	100	100	2.71	3.26

High-angle diffraction measurements (up to 90°) were conducted for silver-containing materials to characterize the small nanoparticles identified by TEM (Figure S6, A). All materials exhibited eight diffraction peaks at around 2θ 27.8, 32.2, 46.2, 54.8, 57.5, 67.5, 76.7, and 85.7°. Comparison of the diffractograms of the silver material with the PDF® database of the International Center for Diffraction Data revealed that they matched the diffractogram of AgCl, indicating that these nanoparticles are composed of AgCl.

Furthermore, the proper encapsulation of phenytoin sodium within the pores of **1-Ag** was validated through an X-ray diffractogram analysis up to 50° (Figure S6B). The diffractogram of **1-Ag@PTN** exhibited multiple peaks of low intensity that corresponded to the drug, and it was compared to a diffractogram of the initial material with phenytoin sodium (MSN@PTN). This confirmed the successful encapsulation of the drug within the pores of **1-Ag**.

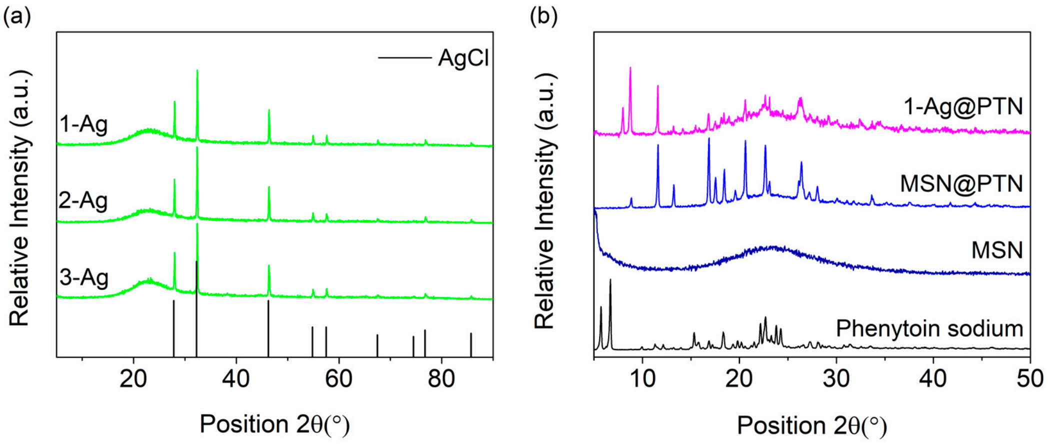

Figure S6. XRD diffractograms of (a) **1-Ag**, **2-Ag** and **3-Ag** with AgCl and (b) **1-Ag@PTN** with MSN@PTN.

1.1.5. FTIR and UV-Vis spectroscopy

The final materials were characterized by infrared (FT-IR) and diffuse reflectance ultraviolet spectroscopy (DR-UV). In the case of infrared spectra (Figure S7) in the starting and final materials the typical O-H vibration bands attributed to the silanol groups and the adsorbed water molecules (between 3500 and 3200 cm^{-1}) and the band corresponding to the deformation vibrations of the water molecules adsorbed on the surface of the material at ca 1625 cm^{-1} can be observed. In addition, the spectra showed a broadband corresponding to the siloxane group (Si-O-Si) at approximately 1100 cm^{-1} and the vibration band of the Si-O bonds, which appeared at about 900 cm^{-1}. In addition to the signals of the silica, a set of less intense bands were observed corresponding to the AP and FQ derivative ligands. Thus, C-H and N-H vibrational bands were recorded between 2700 and 3400 cm^{-1}. Furthermore, a bunch of lower intense bands were observed between approximately at 1350 and 1750 cm^{-1} attributed to C=C and COO or CON signals of FQ fragments. Finally, the **1-Ag@PTN** loaded material (Figure S7A) showed, in addition to the signals mentioned above, a set of signals with higher intensity corresponding to phenytoin sodium encapsulated. These signals were visible between 3200 and 3400 cm^{-1} (corresponding to the N-H bonds), 1720 and 1780 cm^{-1} (attributed to the C=O bonds) and between 700-800 cm^{-1} (C-H bending signals).

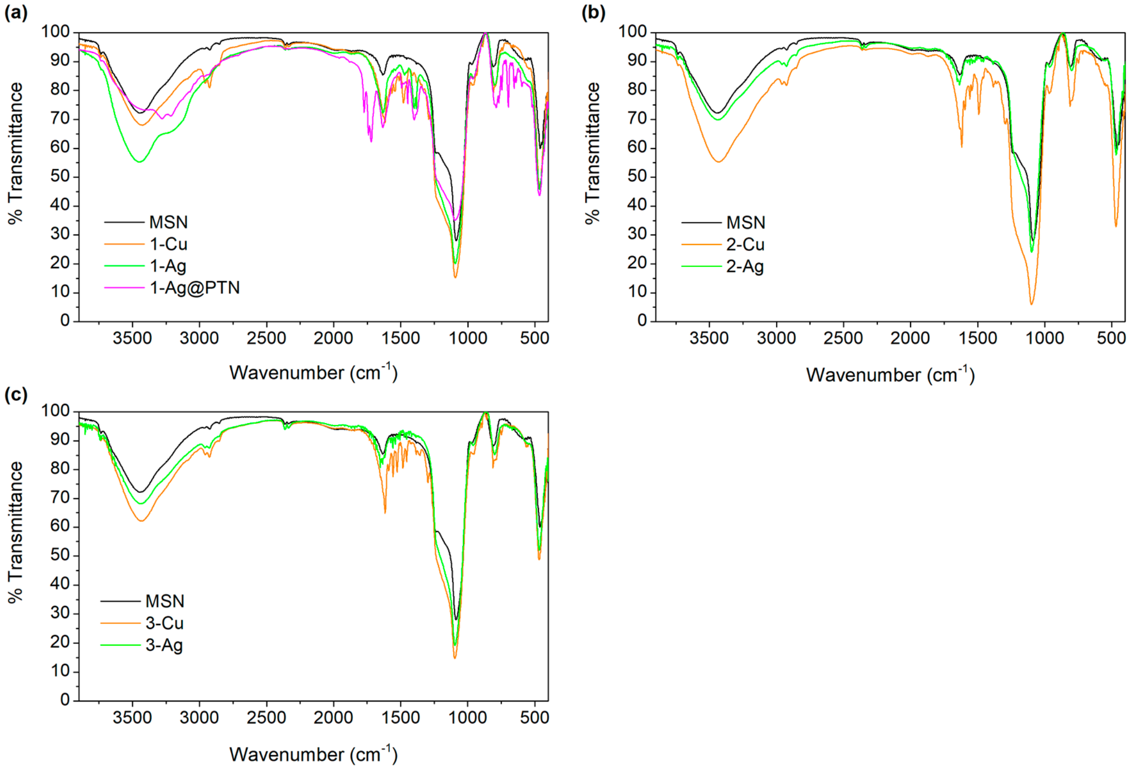

Figure S7. FT-IR spectra of the starting material **MSN** and final functionalized materials based on (a) **1**, (b) **2** and (c) **3**.

In the DR-UV spectra (Figure S8), an intense peak at 220 nm and the bands shown by the materials around 330-340 nm a lower confirmed the incorporation of the AP and

FQ ligands. In addition, the copper systems showed a broad, low-intensity band between 600 and 800 nm corresponding to the d-d transitions of copper. Moreover, the spectra of the final materials with silver showed a broad band or a low-intensity shoulder between 400 and 700 nm, which corresponds to the surface plasmon resonance of silver nanoparticles. Finally, it was observed that the **1-Ag@PTN** material (Figure S8A), in addition to the bands associated with the organic ligands, contained an additional band in the range 220 and 250 nm, as a result of the incorporation of phenytoin sodium into the system.

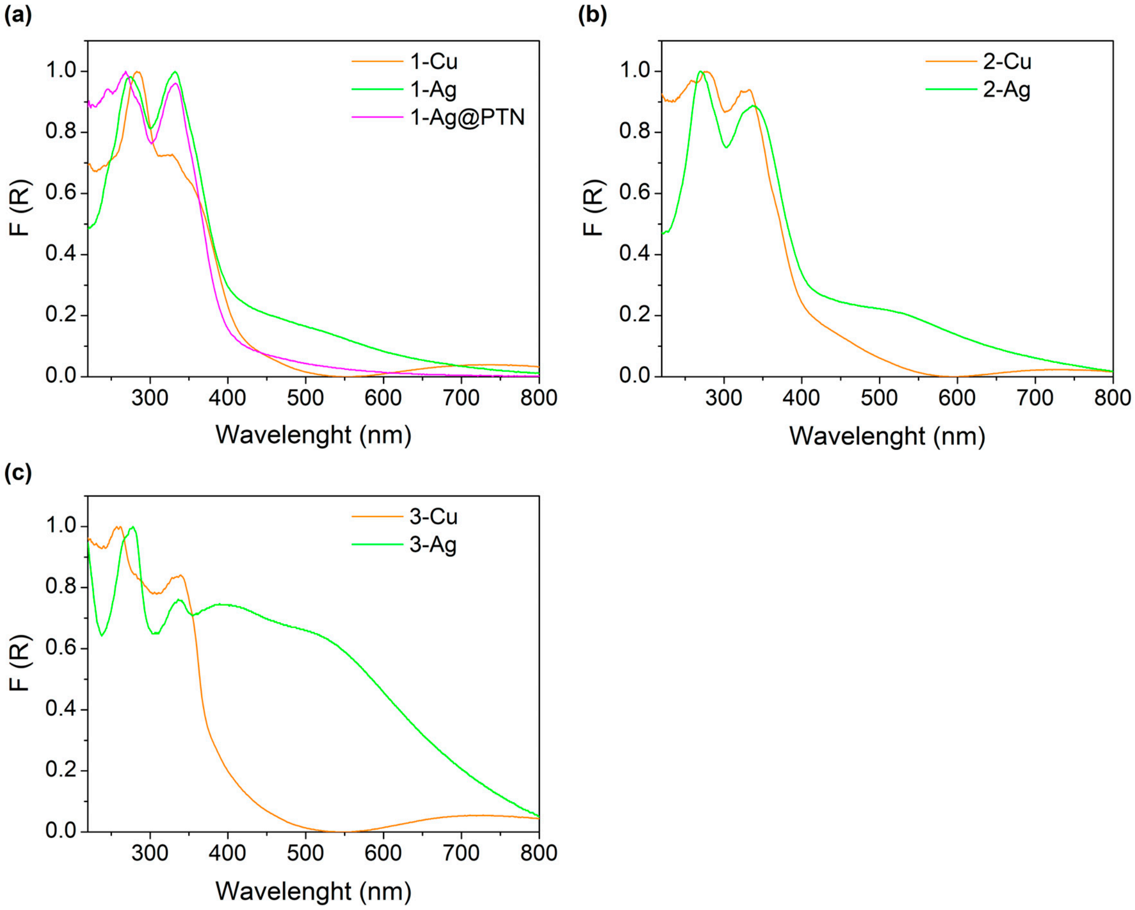

Figure S8. DR-UV spectra of the final materials based on (a) **1**, (b) **2** and (c) **3**.

1.1.6. Solid-state NMR spectroscopy

In order to test the adequate functionalization of the materials with AP and FQ, **1**, **2** and **3** materials were characterized by ^{13}C CP MAS spectroscopy (Figure S9). The spectra showed the signals corresponding to the aliphatic carbons of AP fragment (Si-CH_2-CH_2-CH_2-N), to the cyclopropyl of the fluoroquinolone and to the carbons of free OEt groups between ca. 0 and 55 ppm. Furthermore, a cluster of signals is observed between 100 and 150 ppm due to the aromatic carbons of the FQ ligand. Finally, the spectra showed between 160 and 200 ppm signals corresponding to the C=O and C-F groups of FQ.

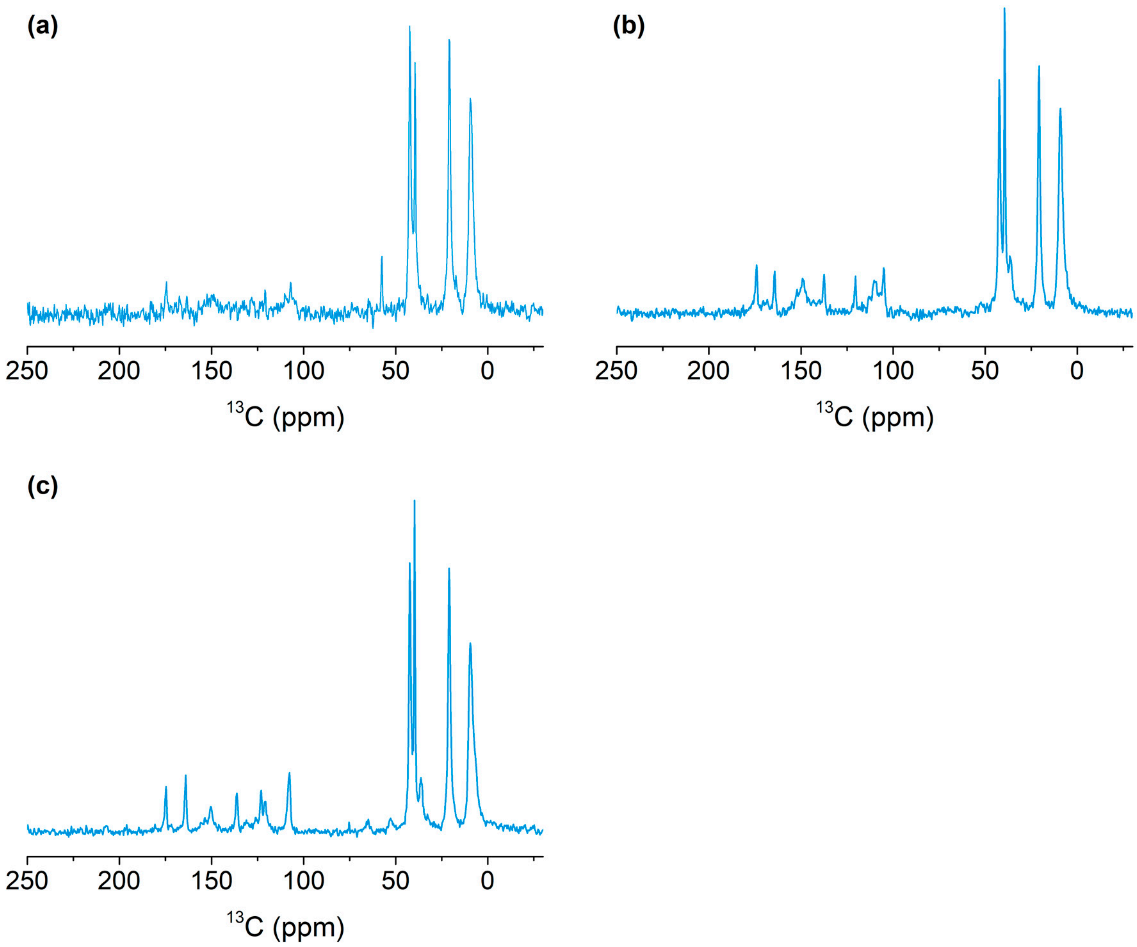

Figure S9. ^{13}C CP MAS NMR spectra of (a) **1**, (b) **2** and (c) **3** materials.

1.2. In Vitro Studies of antibacterial activity

1.2.1. PTN release studies

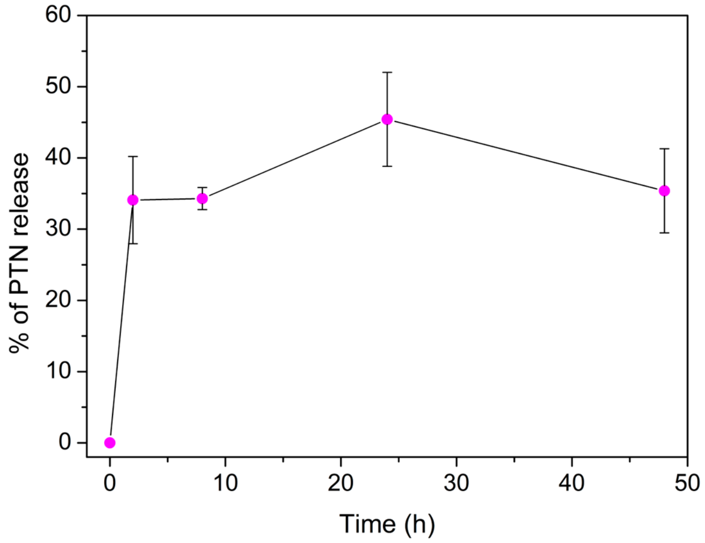

Figure S10. PTN release in **1-Ag@PTN**.
